# Anterior Tooth Width Measurements among Patients in a Tertiary Hospital of Nepal: A Descriptive Cross-sectional Study

**DOI:** 10.31729/jnma.4994

**Published:** 2020-06

**Authors:** Prabhat Shrestha, Sabina Paudel, Madhu Neupane, Suman Lamba

**Affiliations:** 1 Department of Prosthodontics, KIST Medical College and Teaching Hospital, Lalitpur, Nepal; 2 Department of Conservative and Endodontics, KIST Medical College and Teaching Hospital, Lalitpur, Nepal

**Keywords:** *aesthetics*, *dental prosthesis design*, *dimensional measurement accuracy*

## Abstract

**Introduction:**

The knowledge of anterior tooth width and their relationships with each other is essential for any esthetic and prosthodontic rehabilitation. The objective of this study is to measure the width of the anterior teeth of patients coming to a teritary hospital of Nepal.

**Methods:**

This descriptive cross-sectional study was conducted at a tertiary care hospital from 30^th^ September 2019 to 30^th^ October 2019 after receiving ethical clearance from the institutional review committee (reference number: 2076/77/20). Convenient sampling was done. Point estimate at 99% Confidence Interval was calculated along with frequency and proportion for binary data. Data analysis was done in Statistical Package for the Social Sciences version 21.

**Results:**

Out of the 40 participants, the mean width of right and left side of anterior teeth of the maxillary central incisors were 8.62±0.62 mm and 8.65±0.55 mm; maxillary lateral incisors were 6.97±0.74 mm and 7.11±0.78 mm; maxillary canine were 7.81±0.69 mm and 8.15±0.72 mm; mandibular central incisors were 5.37±0.4 mm and 5.43±0.37 mm; mandibular lateral incisors were 5.88±.52 mm and 6.06±0.53 mm; mandibular canine were 6.69±0.55 mm and 6.93±0.7 mm respectively. The difference between the teeth was compared with the central incisors of each side.

**Conclusions:**

Our findings of the average values of the anterior teeth and their difference from the central incisors on each side showed an agreement with the optimal relationships of anterior teeth, with the exception of the maxillary lateral incisors, which were 0.5mm larger than the values of the optimal relationship

## INTRODUCTION

The width of anterior teeth and their relation to one another, play a crucial role in dental prosthesis design, especially when replacing, missing or modifying malformed teeth and in the treatment planning of esthetics rehabilitation where patients desire to improve their smile appearance.^1^

The difference in dimensional measurement accuracy between one tooth to another gives an idea to the prosthodontist and dental laboratory regarding the size of teeth to be replaced or modified during prosthodontic rehabilitation after or during orthodontic treatment or implant placement.^2^ To the best of authors’ knowledge there have not been any studies on individual tooth width and their relationship on Nepalese population till date.

The objective of this study is to measure the width of individual anterior teeth attending various dental departments of KIST Medical College.

## METHODS

A descriptive cross-sectional study was conducted at KIST Medical College and Teaching Hospital, Nepal from 30^th^ September 2019 to 30^th^ October 2019. Ethical clearance approval was received from the institutional review committee (IRC) of KIST Medical College (reference number: 2076/77/20). The data was collected from patients visiting the OPD (outdoor patient department) of the Department of Prosthodontics and the Department of Conservative and Endodontic of KIST Medical College. The study included participants above 18 years with the presence of all maxillary and mandibular anterior teeth. The participants with anterior crowding which prevented visibility of the total mesio-distal width from the labial aspect of anterior teeth, participants who had previously or currently undergoing orthodontic treatment, presence of a fracture or traumatic injury to the anterior teeth and evidence or history of incisal edge/proximal tooth alteration as in i.e. restorative interventions were excluded from the study.

A measuring scale (Isomars, Gridding scale, Y.S International, New Delhi) was customized so that it could be inserted below the teeth and represent a reference for calibrating the measurements in the photographs. A cheek retractor was used to retract the lips and cheeks in the photograph for complete visualization of the tooth and the modified scale was placed below and along the coronal axis of the tooth. A DSLR camera (Sony n200) with a ring flash (Viltrox Macro ring lite JY670) was used to take photographs of the mandibular and maxillary anterior teeth (from central incisor to canine teeth, of each quadrant). The pictures were taken at the right angle of the labial surface of each respective tooth, hence a total of twelve photos were taken of each patient. All the selected participants were informed about the procedure to be performed and written informed consent of the patients was obtained. Convenience sampling was done.

Sample size was calculated as,

n = Z^2^ × (S.D.)^2^/e^2^

   = (2.58)^2^ × (0.6)^2^/(0.25)^2^

   = 38.34

where,

n = sample size
S.D. = standard deviation= 0.6, taken from a previous study^3^
e = margin of error (25 %).
Z = 2.58 at 99% CI.

The sample size was calculated to be 38.34, however, a sample of 40 patients was taken. The photos were opened in Photoshop (Adobe Photoshop CC, 2014) and were enlarged to accommodate the majority of the screen. The scale of the image was used to calibrate the measuring scale of Photoshop (By clicking the image tab^ analysis^set analysisnset measurement scalencustom. The logical length was changed into 10 and the logical unit was kept as mm and 10 mm on the scale in the picture was measured with the ruler tool to calibrate the pixel length). The tooth was aligned parallel to its long axis by using the ruler tool(a line was drawn perpendicular to the long axis of the tooth and straighten layer tab was clicked). Using a pencil tool, vertical lines were drawn on either side of the tooth at the widest mesial and distal point of the tooth. The horizontal distance between the two lines was measured with the ruler tool and the measurement was recorded. This procedure was repeated for each photograph and data was entered in Statistical Package for Social Sciences (SPSS version 21). The mean values and standard deviation and range were derived. The difference of widths of teeth was compared with the central incisors of the respective side after the numerical data were rounded up to the nearest 0.5 mm to make it clinically applicable.^4^

## RESULTS

The mean mesiodistal width and standard deviation of the 40 participants of maxillary; right central incisor (UR_CI)= 8.62±.62, right lateral incisor (UR_LI)= 6.97±.74, right canine (UR_CI)= 7.81±.69, left central incisors (UL_CI)= 8.65±.55, left lateral incisors (UL_LI)= 7.11±.78 and left canine (UL_C)= 8.15±.72 (Table 1).

The mean mesiodistal width and standard deviation of the mandibular; right central incisors (LR_CI)= 5.37±.40, right lateral central incisors (LR_CI)= 5.88±.52, right canine (LR_C)= 6.69±.55, left central incisors (LL_CI)= 5.43±.37, left lateral incisors(LL_ LI)= 6.06±.53 and left canine (LL_C)= 6.93±.70 (Table 2).

The range of the maxillary and mandibular anterior teeth is presented in (Table 1 and Table 2) respectively.

(Tables 3 and 4) show the difference in the width of each tooth with respect to the central incisors on each corresponding side after the numerical data were rounded up to the nearest 0.5 mm to make it clinically applicable^4^.

Among the total participants, 17 (42.5%) were male and 23 (57.5%) were females. The age of the patients ranged from 18 years to 40 years with 70% of participants between 18 to 25 years category.

**Table 1:** Showing the mean, standard deviation, and range of mesio-distal width of maxillary anterior teeth (mm).

	UR CI	UR LI	UR C	UL CI	UL LI	UL C
Mean±SD	8.62±.62	6.97±.74	7.81±.69	8.65±.55	7.11±.78	8.15±.72
Minimum	7.33	5.45	5.91	7.51	5.77	6.33
Maximum	10.06	8.50	9.98	9.71	8.87	9.67

**Table 2:** Showing the mean, standard deviation, and range of mesio-distal width of mandibular anterior teeth (mm).

	LR CI	LR LI	LR C	LL_CI	LL_LI	LL C
Mean± SD	5.37±.40	5.88±.52	6.69±.55	5.43±.37	6.06±.53	6.93±.70
Minimum	4.62	4.73	5.35	4.87	4.81	5.23
Maximum	6.24	7.00	7.93	6.43	7.37	8.52

**Table 3:** Showing the difference in relationship of the mesio-distal width of the right side of maxillary and mandibular anterior teeth with respect to the central incisors (X), when the mesio-distal width is rounded up to the nearest 0.5 mm.

UR CI (X)	UR LI	UR C	LR CI	LR LI	LR C
8.5 mm	X- 1.5 mm	X-1 mm	X- 3 mm	X- 2.5 mm	X- 2 mm

**Table 4:** Showing the difference in the relationship of the mesio-distal width of the left maxillary and mandibular anterior teeth with respect to the central incisors (X), when the mesio-distal width is rounded up to the nearest 0.5mm.

UL CI (X)	UL LI	UL C	LL CI	LL LI	LL C
8.5mm	X-1.5 mm	X-1 mm	X-3 mm	X-2.5 mm	X- 2.5 mm

## DISCUSSION

The maxillary central incisor is the most dominant element in the anterior dental composition,^5^ due to being the widest teeth in the maxillary arch.^6,7^ Dominance appears to be the most important parameter of facial esthetics.^7^ This concept was suggested since back in the 1960s by Zarakani where he improvised that the most important factor in determining esthetics is the width of central incisors, followed by widths of lateral incisors and canine teeth individually.^8^ This is why we have chosen the central incisor as a reference to compare the width of other anterior teeth.

In our study, an easy and practical method of using Adobe Photoshop CC was applied. The width of the teeth was measured by magnifying the size of the images and using a standard protocol. Our method eliminates the stages of impression making and forming a cast which is necessary for measuring the width of teeth by the “gold standard” method of direct caliper measurement.^9^ Another advantage of our technique is it becomes easier to get precise readings of the tooth width as we can magnify the image and maintain the accuracy of measuring scale. Therefore this technique may be more accurate than measuring with the “gold standard” using a measuring gauze on a cast.^10^

Anterior teeth designed by precise planning will give the best esthetic results. Most of the work on anterior tooth measurements are dominated by theories applicable to complete denture work fabrication.^10^ These works are designed to calculate teeth when none of the anterior teeth are present. But a prosthodontist will face a lot of cases where only one or few naturally or congenitally missing teeth, malformed teeth, or dental anomalies are present and their reconstruction has to be done in accordance with the remaining teeth. A few popular concepts like the Golden proportion, Preston proportion, and Recurrent Esthetic Dental (RED) proportion rules have established the ratio of an anterior tooth,^3,11^ but they are applicable only when viewed from the facial aspect.^3^ These concepts are not applicable for the width determination of single tooth replacement as they are not true ratios of individual teeth width. These concepts only accentuate the need of the maxillary arch and neglect the mandibular anterior teeth which may also be more prominent in some patients and play an esthetic role.^12^ Therefore, it is necessary to find the tooth to tooth dimensional width ratios of all anterior teeth. By offering an adequate tooth to tooth relationship, the esthetic result of the oral rehabilitation treatment by the prosthodontist and dental technician can be improved.^5^

There have been very few studies calculating the widths of all mandibular and maxillary anterior teeth and their relations with each other. Many researchers have claimed that there is no universally accepted method to guarantee an adequate tooth-to-tooth relationship that can be used reliably to aid artificial tooth selection, particularly for partially dentate patients.^5^ However D. German et al. published an article, simplifying an optimal formula for calculating the widths of the anterior teeth.^2^ They proposed that the width of the maxillary lateral incisors (U_LI), maxillary canines(U_C), mandibular central incisors (L_CI), mandibular lateral incisors (L_LI), mandibular canines (L_C) should be 2 mm, 1 mm, 3 mm, 2.5 mm and 2 mm respectively narrower than the maxillary central incisors (U_CI). Our study showed similarity to the formula with the exception of only the maxillary lateral incisors, which were 0.5 mm larger than the formula given by the authors. The width may have differed due to measuring techniques or variation of ethnicity.^9,10^

Although our study had a limited number of participants, there were diversities in ethnic groups and age distribution hence this study may represent a tentative picture of the average anterior tooth width measurements of the Nepalese population.

The limitation of the study is that since the study was done in small sample size and convenient sampling was done, hence the further study of a higher level of evidence with a random sampling technique and in a larger setting is recommended.

## CONCLUSIONS

The average values of widths of the anterior teeth derived from this study can be recommended as a template for prosthodontic and esthetic planning in the Nepalese population. Since this is a single institutional study, a larger sample size with diverse ethnic groups is needed to portrait this study as an average in the Nepalese population.

## Conflict of Interest:

**None.**
